# Social Media Interventions Strengthened COVID-19 Immunization Campaign

**DOI:** 10.3389/fped.2022.869893

**Published:** 2022-04-05

**Authors:** Antonio Di Mauro, Federica Di Mauro, Sara De Nitto, Letizia Rizzo, Chiara Greco, Pasquale Stefanizzi, Silvio Tafuri, Maria Elisabetta Baldassarre, Nicola Laforgia

**Affiliations:** ^1^Pediatric Primary Care, National Pediatric Health Care System, Margherita di Savoia (BAT), Italy; ^2^Department of Prevention, Local Health Authority of Bari, Bari, Italy; ^3^Department of Biomedical Science and Human Oncology, University of Bari, Bari, Italy; ^4^Department of Interdisciplinary Medicine, University of Bari, Bari, Italy

**Keywords:** social media, vaccine hesitancy, COVID-19 vaccine acceptance, COVID-19 pandemic, primary care pediatrician

## Abstract

**Background:**

Since The Italian Medicines Agency (AIFA) has recommended the COVID-19 vaccine Comirnaty in children aged 5–11, the immunization campaign faced vaccine hesitancy in parents. Social media are emerging as leading information source that could play a significant role to counteract vaccine hesitancy, influencing parents' opinions and perceptions. Our aim was to evaluate the coverage of the COVID-19 vaccine Comirnaty in a cohort of children aged 5–11 whose families have been counseled to use Social Media to counteract vaccine hesitancy.

**Methods:**

All parents of children aged 5–11 in a primary care setting were instructed by their pediatrician to get accurate information about the COVID-19 vaccine from a *Facebook* page. Active calls to vaccinate children were also scheduled through messaging services *Pediatotem* and *Whatsapp*. Vaccination rates of children in the study were assessed with an electronic database and compared to both regional and national child vaccination rates.

**Results:**

Coverage of 277 children aged 5–11 was analyzed from 16 December 2021 to 31 January 2022. A total of 62.4% (173/277) of enrolled children received the 1st dose of COVID-19 vaccine Comirnaty and 39.7% (110/277) the 2nd dose. Coverage rates were higher compared both to the regional population (1st dose: 48.8%, 2nd dose: 24.6%; *p* = 0.001) and national population (1st dose: 32.1%, 2nd dose: 13.8%; *p* < 0.001).

**Conclusion:**

Increasing vaccine confidence using Social Media interventions have a positive impact on vaccination acceptance of parents.

## Introduction

Since the beginning of the COVID-19 pandemic in Italy, more than two million children and adolescents aged 0–19 years have been infected by the Sars-Cov-2 virus. Of these cases, more than 12,000 children needed hospitalization, around 300 required intensive care, and 39 have died. Pediatric cases represent 20% of the Italian Sars-Cov-2 infections, with an estimated lethality rate of <0.1% (1).

Despite most pediatric cases being milder forms compared to adult ones, growing evidence has shown that a minority might experience a severe multisystem inflammatory syndrome temporally associated with COVID-19 (MIS-C) (2).

Furthermore, there is now mounting evidence of persisting symptoms in children following acute Sars-Cov-2 infection, which has been named Long COVID, and studies that highlight how lifestyle changes during the COVID-19 pandemic have had a significant psychological impact on the pediatric population (3, 4).

For all these reasons, since a vaccination regimen of two doses of the Comirnaty vaccine was found to be safe, immunogenic, and efficacious in children 5–11 years of age (5), the Italian Medicines Agency has recommended an extension of Comirnaty vaccination to this pediatric population.^1^

Parents' attitude toward vaccine use is a key factor affecting children's immunization programs and vaccine hesitancy is a known threat to global health (6–8). In this scenario, social media are emerging as a leading information source that could play a significant role in increasing or mitigating vaccine hesitancy, influencing the opinion and perceptions of parents (9–11).

The primary aim of this study is to evaluate coverage of COVID-19 vaccination in a cohort of children 5 to 11 years of age, whose families have been counseled to use Social Media to counteract vaccine hesitancy.

The secondary aim is to compare vaccination rates of the interventional cohort with those of regional and national pediatric populations of the same age range.

## Methods

### Population and Setting

We performed a prospective study in a primary care setting to evaluate coverage of COVID-19 vaccination in a cohort of children aged 5–11 years from 16 December 2021 to 31 January 31 2022.

Children of the study cohort were followed at Pediatric Primary Care Office (PPCO) in Margherita di Savoia (BAT, Apulia, Italy) which offers primary pediatric care to 651 children from birth through adolescence.

According to the national guidelines, the regional Apulian government offered two 10-μg doses of the Cominraty vaccine administered 21 days apart, free of charge, to children aged 5–11 years.

Furthermore, the Regional Apulian Covid-19 Immunization Campaign started an active collaboration between the Primary Care Pediatricians, the Local Health Organizations (Public Health Departments), and the Regional School Offices, focused on collective vaccination process management. In fact, the Local Health Authority set up a COVID-19 Vaccine Pediatric Hub in a school gym in Margherita di Savoia, driven by five local primary care pediatricians.

### Interventions

The primary care pediatrician of Margherita di Savoia has managed a Social Media-based strategy to counteract COVID-19 vaccine hesitancy in parents.

Through a professional *Facebook page* (https://www.facebook.com/antoniodimauropediatra) with a fanbase of over 50,000 users, he posted, on a regular basis, official and certified messages on health, vaccine-related scientific data, info-graphics, useful information, and videos from institutional pages, such as that of the *Italian Society of Pediatrics* (https://www.facebook.com/societaitalianadipediatria).

Other Facebook posts were arranged into short, easy-to-read paragraphs, discussing risks and benefits of vaccines, and news on pediatric COVID-19 and its management. The pediatrician certified the validity and trustworthiness of the material posted. Posts used in the study are easily accessible and manageable to allow replication studies in previously cited Facebook Pages.

All parents were allowed to post comments and questions and to get answers from the pediatrician in a public dashboard, thus overcoming the obsolete one-way communication typical of traditional media.

In the study period, 84 posts were published with a total of 462,883 interactive visualizations, 34,398 likes, 5.450 shares, and 5,707 comments. The total estimated coverage of the Social Network activity was of 1,811,560 Facebook users reached. All these data were extracted by Facebook Insight.

Furthermore, four active calls to vaccinate children, on dedicated open days, was also scheduled through messaging services *Pediatotem* and *Whatsapp*.

### Statistical Data

The list of children aged 5–11 followed in the Margherita di Savoia PPCO was obtained by a regional database (EDOTTO). For each children doses were registered in a standardized form, obtained through the Apulian Regional Vaccination Register (GIAVA).

Coverages of Margherita di Savoia PPCO were compared with that of the Apulian and Italian pediatric population of 5–11 years of age. All data were extracted on 31 January 2022 and analyzed by STATA MP12 software. Categorical variables were expressed as proportions. The Chi-square test was used to compare proportions. A *p* < 0.05 was considered as significant.

## Results

Of the 651 children followed at Margherita di Savoia PPCO, 277 were aged 5–11 years (42.5%).

From 16 December 2021 to 31 January 2022, 173 (62.4%) of enrolled children received the 1st dose of Comirnaty and 110 (39.7%) the 2nd dose.

**Figure 1:**
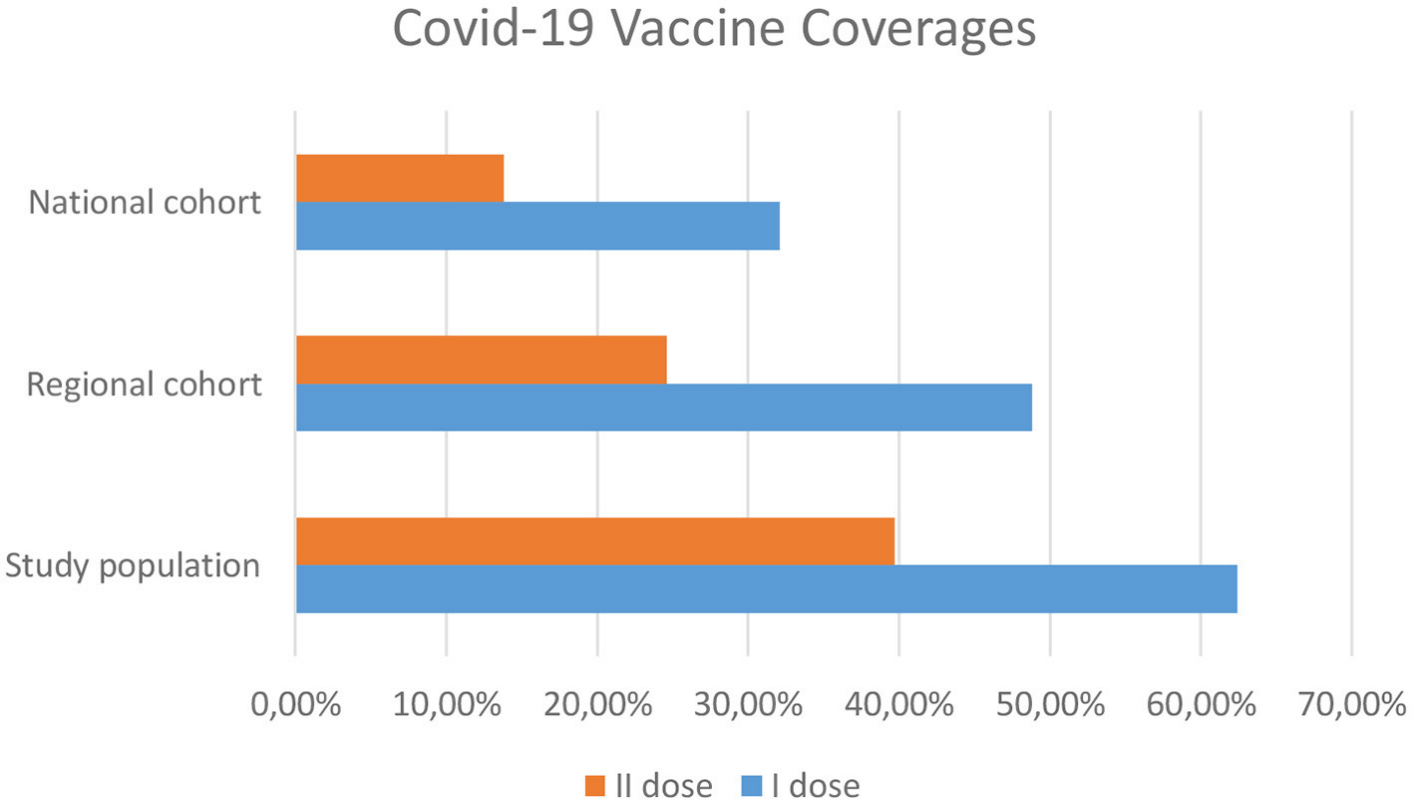
A significantly higher coverages were observed in the study population compared to both regional (Table 1) and national ones (Table 2).

On the same day, the regional child population of 5–11 years exhibited an immunization rate of 51 and 25.1% for the 1st and 2nd dose of the Comirnaty vaccine, respectively. COVID-19 vaccine coverage decreased to 32.1 and 13.8% for the 1st and 2nd dose respectively of the Comirnaty vaccine when the entire Italian child population aged 5–11 was analyzed (Figure 1).

## Discussion

Our study shows that COVID-19 vaccination coverage in pediatric patients is higher when parents have been subjected to social media-based interventions, compared to the general pediatric population (Tables 1, 2).

**Table 1:** Differences in vaccine coverages between study cohort and regional population.

	**Study population**	**Regional population**	***P***
I dose	62.4% (173/277)	48.8% (117.366/240.444)	0.001
II dose	39.7% (110/277)	24.6% (59.289/240.444)	0.001

**Table 2:** Differences in vaccine coverages between study cohort and national population.

	**Study population**	**National population**	***P***
I dose	62.4% (173/277)	32,1% (1.175.365/3.656.069)	<0.0001
II dose	39.7% (110/277)	13.8% (505.931/3.656.069)	<0.0001

It is of note that Apulian coverages are higher compared to the Italian pediatric 5–11 years population as an effect of efficacious vaccination strategy adopted at a regional level. Apulia scores first in the national ranking for vaccination in the 5–11 age group, with 48.8%, 16.7% points above the national average. These data confirm the fundamental active collaboration between Primary Care Pediatricians, Local Health Organizations (Public Health Departments), and Regional School Offices.

We believe that the Social Media-based vaccination strategy enforced the regional campaign, overcoming parental COVID-19 vaccine hesitancy, with well-structured and continuous counseling activity on Social Media that supports the final parental vaccination decisions.

Our results are different, but very promising, from the growing literature on parental COVID-19 vaccine hesitancy (12–14). On the other hand, another study suggested how an actively and directly neonatologists' interaction on Social Media can improve vaccine acceptance in preterm infants' parents after hospitalization (15).

With the increasing use of the internet in the last decades, social media became an attractive platform to promote a healthy lifestyle (16). In this scenario, a new form of social media celebrity, defined as “influencers”, has emerged as credible in specific topic areas and so are followed by a fanbase, with the real possibility to promote healthy lifestyle behaviors.

The Italian Paediatric Society has previously promoted healthy lifestyle campaigns, engaging pediatricians as “influencers”, demonstrating how social media increased families' interaction with correct information, thus contrasting the spreading of fake news (17). However, in this previous pilot study, they did not analyze the effects of influencers' social media interventions on the health of children and adolescents.

To the best of our experience, this is the first study that analyses the effects of social media interventions directly on an Italian pediatric cohort. Our high vaccination coverage demonstrated how social media could be a very useful partner for vaccination campaigns, especially in a pediatric primary care setting.

Primary Care Pediatricians' counseling, online contributions, opinions, and posts could be essential to recovering hesitant parents, considering both the relationship of trust with the families and their credible reputation, known in local setting due to the capillary network of Italian PCCO (18).

For such reasons, according to others, we encourage Primary Care Pediatricians' active participation in social media communication (19).

The strengths of our study were the social media strategy organized by a trained pediatric influencer and the accuracy of data extracted from a computerized surveillance system (GIAVA). There are also some limitations. First, we are aware that further RCT trials are needed to confirm our data because we compared our cohort to general regional and national populations, with no randomized intervention and a short enrolment period. Secondly, participants had unlimited access to social networks, but we cannot know how they spent time, participate and have effective dialogic communication with posts we published on the Facebook page. RCT trials with specific surveys among parents may better clarify the role of social media interventions. Finally, the trial was conducted in a single PPCO, and in a specific region with a very effective protocol for vaccination and our results could not be applied to other Local Health Systems without the same availability of vaccine services.

## Conclusions

A scientific-based use of social media, with effective dialogic communication and interpersonal influence, could be considered as useful partners in vaccination campaigns to positively influence parental vaccine acceptance. In our experience, the trusting Pediatrician-Family-Patient relationships built *via* the social web strengthened the effective collaboration between Primary Care Pediatricians and the Local Health Authorities.

## Data Availability Statement

The raw data supporting the conclusions of this article will be made available by the authors, without undue reservation.

## Ethics Statement

Ethical review and approval was not required for the study on human participants in accordance with the local legislation and institutional requirements. Written informed consent from the participants' legal guardian/next of kin was not required to participate in this study in accordance with the national legislation and the institutional requirements.

## Author Contributions

AD planned the study, coordinated the study, and wrote the first draft of the manuscript. FD, LR, and SD examined the data from national and regional dataset. CG and PS explored the literature and performed statistical analysis of data. NL, MB, and ST revised the final manuscript. All authors have read and approved the final manuscript.

## Conflict of Interest

The authors declare that the research was conducted in the absence of any commercial or financial relationships that could be construed as a potential conflict of interest.

## Publisher's Note

All claims expressed in this article are solely those of the authors and do not necessarily represent those of their affiliated organizations, or those of the publisher, the editors and the reviewers. Any product that may be evaluated in this article, or claim that may be made by its manufacturer, is not guaranteed or endorsed by the publisher.

## Footnotes

^1^ https://www.aifa.gov.it/documents/20142/1123276/Parere_CTS_Comirnaty_5-11_01.12.2021.pdf

## Abbreviations

PPCO: Pediatric Primary Care Office.
